# Intra-Seasonal Flexibility in Avian Metabolic Performance Highlights the Uncoupling of Basal Metabolic Rate and Thermogenic Capacity

**DOI:** 10.1371/journal.pone.0068292

**Published:** 2013-06-28

**Authors:** Magali Petit, Agnès Lewden, François Vézina

**Affiliations:** 1 Département de biologie, chimie et géographie, Université du Québec à Rimouski, Rimouski (QC), Canada, Groupe de recherche sur les environnements nordiques BOREAS, Rimouski, Québec, Canada; 2 Centre d’Etudes Nordiques, Québec (QC), Canada, Centre de la Science de la Biodiversité du Québec, Montréal, Québec, Canada; The University of Wollongong, Australia

## Abstract

Stochastic winter weather events are predicted to increase in occurrence and amplitude at northern latitudes and organisms are expected to cope through phenotypic flexibility. Small avian species wintering in these environments show acclimatization where basal metabolic rate (BMR) and maximal thermogenic capacity (M_SUM_) are typically elevated. However, little is known on intra-seasonal variation in metabolic performance and on how population trends truly reflect individual flexibility. Here we report intra-seasonal variation in metabolic parameters measured at the population and individual levels in black-capped chickadees ( *Poecile* *atricapillus* ). Results confirmed that population patterns indeed reflect flexibility at the individual level. They showed the expected increase in BMR (6%) and M_SUM_ (34%) in winter relative to summer but also, and most importantly, that these parameters changed differently through time. BMR began its seasonal increase in November, while M_SUM_ had already achieved more than 20% of its inter-seasonal increase by October, and declined to its starting level by March, while M_SUM_ remained high. Although both parameters co-vary on a yearly scale, this mismatch in the timing of variation in winter BMR and M_SUM_ likely reflects different constraints acting on different physiological components and therefore suggests a lack of functional link between these parameters.

## Introduction

With global changes comes a higher frequency of unpredictable weather events [1]. These short-term environmental fluctuations can affect animal species through changes in demography, phenology [2] and genetic variability [3], for example by disturbing the timing of reproduction [4,5] or by favouring genotypes that produce flexible rather than stable phenotypes [3]. This may be particularly important for northern latitude species where warming is accelerated relative to lower latitudes [6], especially in winter [7], and where the occurrence and amplitude of short term stochastic events are predicted to increase [6]. Phenotypic flexibility, the rapid and reversible transformations of phenotypic traits that allow individuals to adjust their behaviour and physiology to predictable or stochastic changes in the environment [8], should provide a certain capacity to buffer these variations [3]. However, in natural settings, little is known on how animals adjust their phenotype to intra-seasonal changes in ecological conditions [9,10].

Winter at northern latitudes is typically considered a challenging season for resident bird species [11]. Since they remain active throughout the cold season, low ambient temperatures force these animals to increase energy expenditure for thermoregulation [12,13] while short days, snow and ice cover may reduce foraging time and food availability [14,15]. In small bodied species, thermoregulatory constraints are exacerbated because of their large surface area relative to volume, which increases heat loss [16], in addition to their limited ability to carry thick insulative plumage. Small birds therefore use physiological adjustments to improve cold tolerance [12,17] and their chances of survival. Seasonal acclimatization is typically associated with a winter increase in metabolism visible in parameters such as basal metabolic rate (BMR; physiological maintenance cost) and summit metabolic rate (M_SUM_; maximal thermogenic capacity) [12,17,18]. However, although cold acclimatization has been investigated for decades [19], most field studies lack the required temporal resolution to address questions regarding individual physiological adjustments in response to intra-seasonal variations in ambient conditions [9,20]. Studies typically compare phenotypic traits such as BMR and M_SUM_ on a seasonal basis, comparing values from individuals captured in winter with data collected on different individuals captured in summer [12,13,17,18,21].

To face stochastic winter conditions, small birds could use two mutually non-exclusive strategies. First, they could maintain a constant and elevated winter metabolism. This strategy would increase survival during extreme thermal events but it would also presumably be associated with high and often unnecessary maintenance costs. Second, birds could use phenotypic flexibility to rapidly adjust their physiology to prevailing conditions. In this situation, however, individuals may have to invest considerable resources in physiological readjustments (e.g. adjustment in organ size [22]). As far as we know, only two studies have provided data on intra-seasonal metabolic changes in response to winter climate variations in small free-living birds [10,23]; both of these reflected a phenotypic flexibility strategy. Swanson and Olmstead [10] observed that cold ambient temperature was associated with elevated metabolic performance in dark-eyed juncos ( *Junco* *hyemalis* ), black-capped chickadees ( *Poecile* *atricapillus* ) and American tree sparrows ( *Spizella* *arborea* ). This was visible at the population level on a between-year timescale for BMR and M_SUM_ and on a between-month (i.e. intra-seasonal) timescale for M_SUM_ (there were not enough data for testing the effect on BMR). Furthermore, ambient temperature preceding measurements appeared to affect metabolic performance within relatively short periods, between one and 30 days, depending on the species. Similar findings were reported by Broggi et al. [23] in great tits ( *Parus* *major* ) where BMR changed throughout winter and responded to ambient temperature averaged over the preceding week.

If ambient temperature exerts such a proximal effect on winter metabolic performance, one would therefore expect adjustments in metabolic parameters over the course of the season, with peak capacity observable at the coldest time of winter. However, data on the shape of metabolic transformations within seasons are lacking, albeit being called for [9,20]. Furthermore, although seasonal changes in metabolic performance are interpreted as a clear and evident example of phenotypic flexibility in response to winter constraints [24], within individual data to support this statement are still lacking for free-living wintering birds. Our understanding of intra-seasonal and intra-individual variation in metabolic performance of birds wintering at northern latitudes therefore remains poor.

Here we report results of a study where we followed intra-seasonal changes in metabolic performance over two consecutive winters in a population of black-capped chickadees from eastern Canada. We measured changes in BMR to assess variations in physiological maintenance costs and we measured changes in M_SUM_ to follow adjustments in winter thermogenic capacity. These measurements were also performed in August of each year to obtain a summer reference point for comparison. Our first objective for this part of the study was to determine patterns of variation in BMR and M_SUM_ within winter. We expected a gradual increase in metabolic performance beginning in autumn to reach a peak at the coldest of winter (i.e. January–February), followed by a gradual decline to reach summer values [23]. Our second objective was to confirm that these patterns were also visible within individuals and therefore confirm that observations at the population level reflect individual phenotypic flexibility.

Previous studies suggested a functional link between BMR and M_SUM_ [10,25,26] but some evidence rather suggests that these variables reflect physiological systems acting independently [20,27,28]. BMR would mainly reflect energy requirements of internal organs [18,29,30] while M_SUM_ would reflect the size of muscles involved in active shivering [21,31,32]. Given recent contrasting findings, including in our own model species [28,33], we also had an interest in testing the relationship between BMR and M_SUM_ with an extensive dataset.

Metabolic expansibility (ME), the ratio of maximal over minimal metabolic rates (M_SUM_ / BMR) [15,26,28], is interpreted as the capacity of an organism to increase its level of heat production for a given size of metabolic machinery [12,34]. Therefore, variations in ME should also be a useful variable to evaluate co-adjustment of physiological maintenance costs and thermogenic capacity. We thus report inter- and intra-individual variation in ME throughout winter.

To meet our objectives, 228 individuals were captured during winters 2010 and 2010-11 and had their metabolic performance measured within the following 24h. Of this number, 56 individuals were recaptured and remeasured between one and five times within a same winter.

## Materials and Methods

### (a) Capture and handling

This study was carried out within the Forêt d’Enseignement et de Recherche Macpès, Québec, Canada (48° 30’ N, 68° 52 W) between January and March 2010 (n = 56) and from October 2010 to March 2011 (n = 149). Data from summer individuals were collected in August 2010 (n = 12) and 2011 (n = 11). To attract chickadees and facilitate capture, 18 feeding stations were set up within the forest with an average distance between stations of 1.9 km (see 30,32 for a description of the stations). Feeders were regularly filled with black sunflower seeds. On capture days, feeders were removed and homemade potter traps (15 cm x 15 cm x 15 cm) baited with seeds were placed on a tray installed on a wooden fence pole. All birds were caught during morning (between 08:00 and 13:00) and removed from traps within one minute of capture. Weather stations in the forest [35] recorded temperature data allowing us to track ambient temperatures over the two years of the study.

Birds caught for the first time were banded with a USGS numbered metal band as well as a unique combination of three plastic color bands to allow further identification from a distance. For each capture, birds were first weighed then had the length of their beak, head plus beak, tarsus, tail and wing measured [36]. Following these measurements and depending on capture success, up to four birds per day were brought to the field station for metabolic measurement.

### (b) Ethics statement

All bird manipulations were approved by the animal care committee of the Université du Québec à Rimouski (CPA-37-09-68) and have been conducted under scientific and banding permits from Environment Canada - Canadian Wildlife Service (Permit Number: 10704H).

### (c) Respirometry

Once at the field station, birds were maintained in separate cages (39 cm x 43 cm x 31 cm) supplied with food (sunflower seed) and water *ad libitum* until measurements were made. Cages were kept in a room receiving natural light through a window and maintained quiet to avoid disturbance. At around 13: 00, we began M_SUM_ trials by measuring two birds in parallel using Fox, Box oxygen analyzers (Sable Systems, Las Vegas, NV, USA). This was followed by a second trial on the remaining two birds, which began before 15: 00. Briefly, birds were first weighed (± 0.1 g) and body temperature was measured with a thermocouple reader (Omega model HH-25KC, NIST-traceable, Omega, Montréal, Qc, Canada) using a copper-constantan thermocouple inserted into the cloacae approximately 10 mm deep. Then, birds were put in a stainless steel metabolic chamber fitted with a perch and were exposed to helox gas (21% oxygen, 79% helium) using an average flow rate of 1109 ml.min^-1^ controlled by mass flow valves (Sierra Instruments, Side-Trak® Model 840 (Monterey, CA, USA)). We recorded oxygen consumption of each bird using a sliding cold exposure protocol [37] with a decrease in ambient temperature of 3°C every 20 minutes, starting at 0°C in winter and at 6°C in summer. We ended the trials when birds became hypothermic, which was easily identifiable in real time as a steady decline in oxygen consumption for several minutes. Body temperature was measured again immediately after taking birds out of their chambers. We assumed a bird had reached its M_SUM_ when body temperature after a trial was ≤ 38°C [38] (mean body temperature after M_SUM_ measurement = 33.6 ± 0.2°C). Data from individuals showing a body temperature above this threshold were discarded. Birds were weighed again after measurements and the average body mass was used for the M_SUM_ analysis. Birds were then brought back to their cage with food and water *ad libitum* until BMR measurement starting at 19: 00.

Each day, all four birds had their BMR measured simultaneously overnight (from 19: 00 to 06:00). Individuals were maintained at 30°C throughout the trial (within the thermoneutral zone for this species [39]) and received a constant flow of air. Birds were weighed before and after measurements and average mass was used in BMR analyses.

Oxygen analyzers were adjusted each day to 20.95% using CO_2_-free dry air and mass flow valves were carefully calibrated for air and helox using a bubble-O-meter (Dublin, OH, USA) once per winter. Metabolic rate calculations were done with ExpeData software, v 1.2.6 (Sable Systems, Las Vegas, NV, USA). M_SUM_ and BMR calculations were based respectively on the highest and lowest averaged 10 minutes of oxygen consumption per measurement sequence according to Lighton’s equation 10.1 [40]. The instantaneous measurement technique [41] was used for M_SUM_ while BMR was calculated using the steady state approach. The duration of BMR trials (around 11 hours) insured that birds were post-absorptive at time of BMR measurement (which was obtained after 6 h 40 min ± 8 min of recording on average). Since wintering birds use mostly lipids as substrate for shivering [15,42,43], we estimated energy consumption using a constant equivalent of 19.8 kJ.L^-1^ O_2_ and converted to Watts [44]. After BMR measurements, birds were put back in their cage with access to food and water until release at capture site around 2 hours later.

### (d) Sexing individuals

130 individuals (56 females and 74 males) caught for this study were sexed by PCR [45] or dissection. We determined sex of the remaining 98 birds using their morphometric data in a discriminant analysis [46]. Overall, 85 individuals were identified as females, 107 as males and 36 birds remained undetermined.

### (e) Statistical analysis

#### Inter-seasonal variation

We first studied inter-seasonal variation in body mass and metabolic performance. To do so, we used a linear mixed effect model (LME) to test for effects of “year”, “season” (winter or summer), “sex” (male, female or undetermined) and interaction term “year*season”, using bird ID as random parameter, on whole BMR, M_SUM_ and ME. We also included the variable “relative time of capture” (time since sunrise / day length, hereafter “time of capture”) for body mass analyses. We then used the same model including body mass as a covariate to analyse variations in mass-independent metabolic performance.

#### Intra-seasonal variation

To study intra-seasonal variation (i.e. within winter) in parameters of metabolic performance, we used the same model but this time testing for the effects of “year”, “month”, “sex” and the interaction term “year*month”, again using bird ID as random parameter. “Time of capture” was also considered when analysing variations in body mass. To study effects on mass-independent variables we repeated the analyses including body mass as a covariate.

We saved residuals from those LMEs to study relationships between whole and mass-independent winter BMR and M_SUM_.

Variables were removed from models when non-significant and results from final models are presented. In cases where the interaction term year*month was significant, we ran separate analyses by year. We used Tukey tests to investigate differences between months and between sexes. Analyses on metabolic parameters showed the same final patterns whether or not body mass was included as a covariate. We therefore present results for mass-independent BMR, M_SUM_ and ME. Data for these variables without mass corrections are available in the Supporting Information files (table S1-S2). In all cases, residuals were tested for normality using the one sample Kolmogorov-Smirnov test. Population data are presented as least square means ± s.e.m., and intra-individual data are shown as predicted values (original values corrected for the effects that were found significant at the population level).

## Results

### (a) Temperature

During the first winter of the study (2009-2010), chickadees experienced mean daily temperatures below 0°C from December to March (table 1). The coldest average temperature was recorded in December, and the lowest minimal temperature was measured in February. During the second year (2010-2011), mean temperatures fell below 0°C from November to March and reached their coldest mean ambient and minimal values in January (table 1). However, the following months remained relatively cold as minimal temperatures changed by less than one degree in the next two months. Overall, the second winter of the study was colder than the first and had more months with minimal temperatures below -25°C.

**Table 1:** Monthly minimal, mean and maximal temperatures (° C) recorded by weather station within the study area.

	2009/2010			2010/2011			
	min	mean	max	min	mean	max
October	-6.8	3.1	13.5	-3.2	5.7	19.3
November	-7.3	2.2	13.5	-12.6	-1.2	13.5
December	-20.3	-7.3	2.1	-16.4	-3.6	11.7
January	-21.1	-6.3	8.3	-26.8	-11.4	2.0
February	-24.7	-5.7	6.5	-26.3	-11.2	3.1
March	-15.8	-1.9	9.6	-26.8	-5.4	8.4
August	5.6	18.2	30.7			

(Data for August 2011 not available)

### (b) Inter-seasonal variation in body mass and metabolic performance

Peak values in average metabolic performance were recorded in February for both years (see below). We therefore calculated inter-seasonal variation in body mass and metabolic parameters between peak of winter and summer using values measured in February and August.

**Figure 1:**
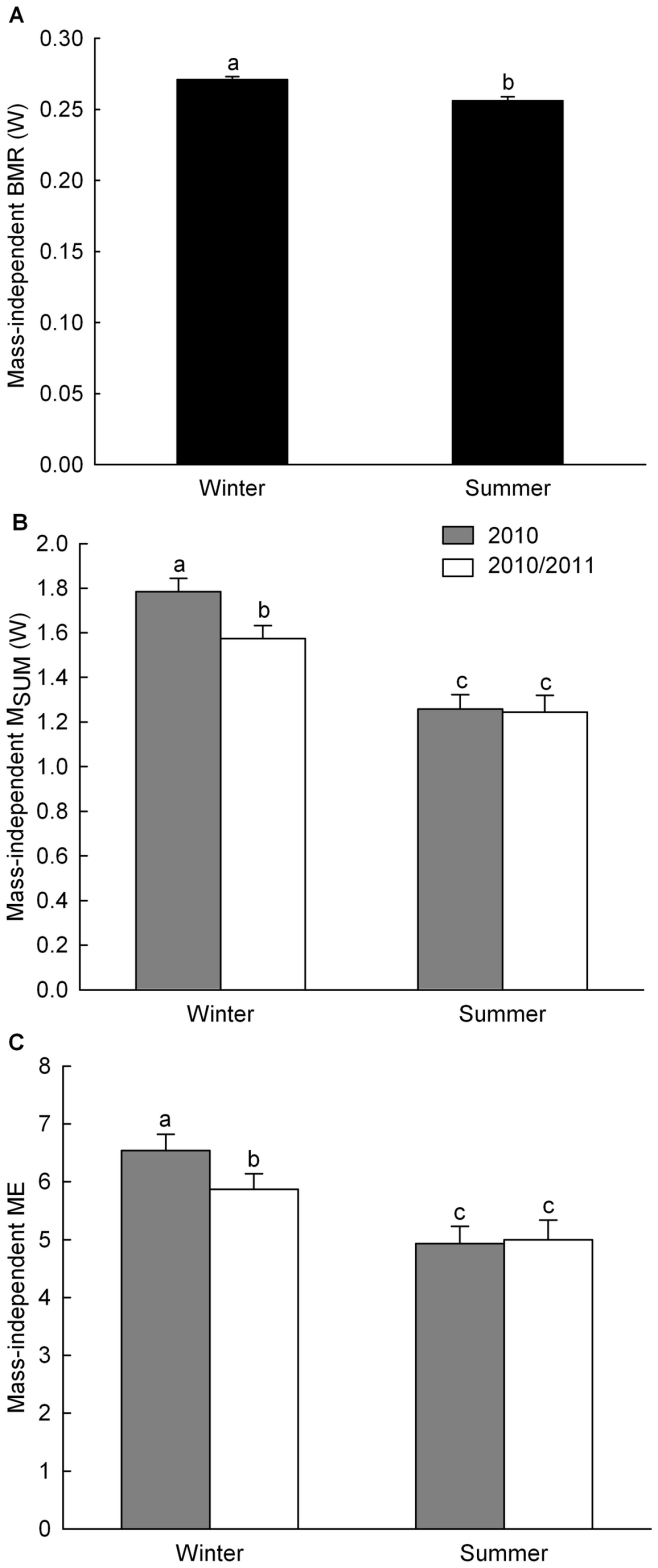
Inter-seasonal variation of mass-independent BMR, M_SUM_ and ME. Data are least square means for BMR (A), M_SUM_ (B) and ME (C) controlling for year, season, sex and body mass with bird ID as random parameter. Different letters indicate significant difference between seasons.

Average body mass did not vary between years or seasons but positively varied with time of capture (F_1,70_ = 11.2, p < 0.01). Males were also on average 10.5% heavier than females (sex: F_2,112_ = 59.8, p < 0.0001, males: 12.06 ± 0.07 g; females: 10.91 ± 0.08 g, undetermined 11.31 ± 0.10 g, Tukey: p < 0.0001). BMR, M_SUM_ and ME were all influenced by body mass (BMR: F_1,112_ = 32.5, p < 0.0001; M_SUM_: F_1,109_ = 29.6, p < 0.0001, ME: F_1,98_ = 5.0, p < 0.05). Mass-independent BMR was 5.9% higher in winter relative to summer (season: F_1,116_ = 16.5, p < 0.0001, Figure 1A) while average values for mass-independent M_SUM_ were 34.2% higher at the peak of winter relative to August (season: F_1,115_ = 135.5, p < 0.0001). However, this latter effect depended on the year (year*season: F_1,117_ = 7.2, p < 0.01). Mass-independent M_SUM_ was 13.4% higher in the first winter relative to the second, which led to a M_SUM_ being 41.9% higher than in summer during the first year compared to a 26.4% seasonal difference during the second year (Tukey: p < 0.0001 in all cases) (Figure 1B). Mass-independent ME was 25.0% higher in winter (6.2 x BMR) than in summer (5.0 x BMR, season: F_1,111_ = 56.9, p < 0.0001) and this effect was also dependent on the year (year*season: F_1,109_ = 5.2, p < 0.05). Mass-independent ME was 11.4% higher in the first winter than in the second with a ME 32.7% higher relative to summer in the first year compared to a 17.4% difference between winter and summer during the second year (Tukey: p < 0.0001 in all cases) (Figure 1C).

### (c) Intra-seasonal variation of body mass and metabolic performance

**Figure 2:**
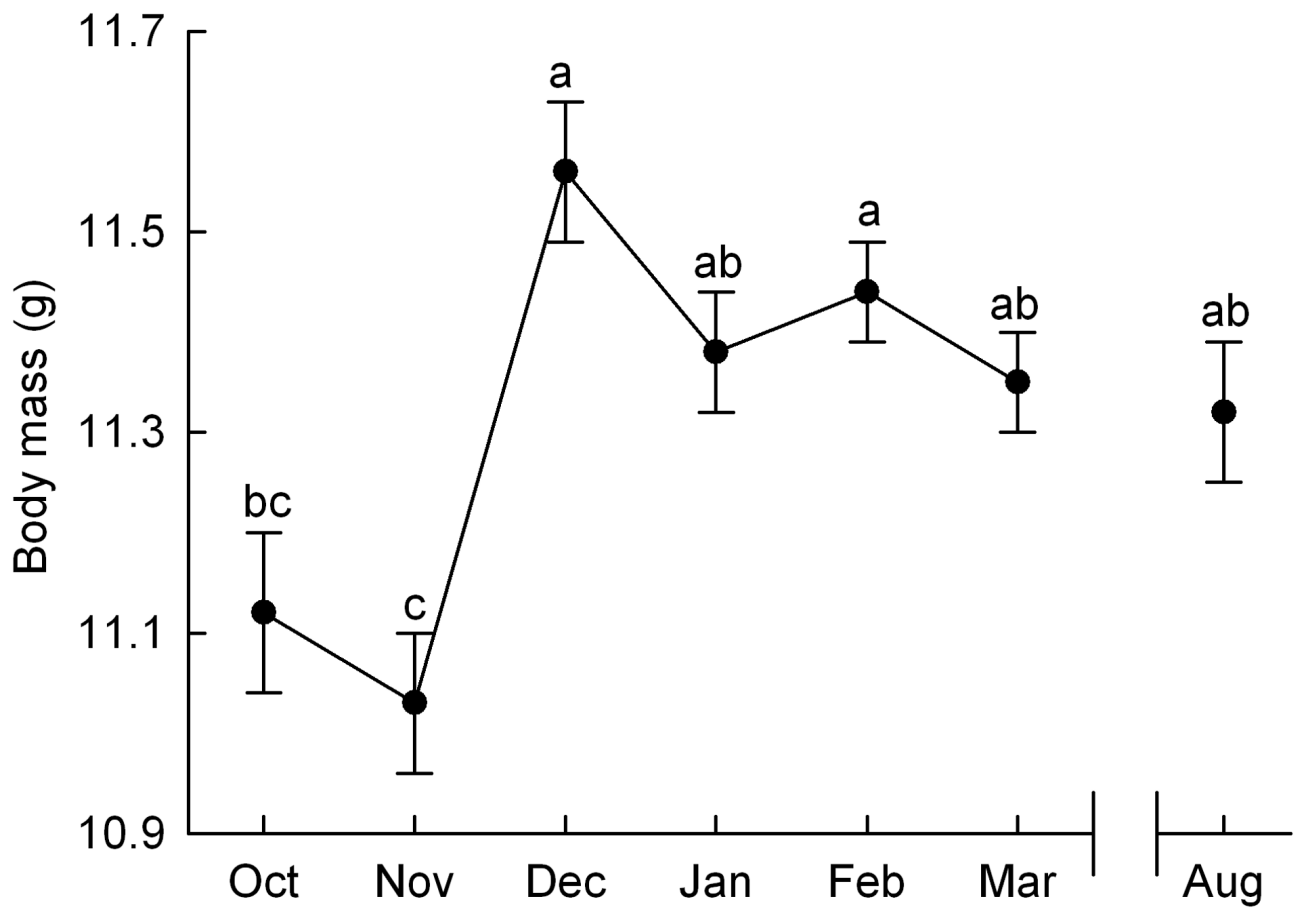
Intra-seasonal changes in body mass. Data are least square means of body mass controlling for year, month, time of capture, sex with bird ID as random parameter. Different letters represent significant difference between months.

As for the inter-seasonal analyses, we found no year effect on average body mass in wintering black-capped chickadees. However, body mass varied over time within winter (month: F_6,212_ = 8.3, p < 0.0001) increasing by 4.0% between October and December (Tukey: p < 0.01) and remained constant until August (Figure 2, Tukey: p = 0.07). Mass also differed between sexes (F_2,218_ = 146.8, p < 0.0001) with males being on average 11.5% heavier than females (males: 12.00 ± 0.05 g; females: 10.76 ± 0.06 g, undetermined 11.18 ± 0.07 g, Tukey: p < 0.0001) and, as these birds are fattening up on a daily basis [35,47], body mass was positively affected by time of capture (F_1,225_ = 57.1, p < 0.0001).

**Figure 3:**
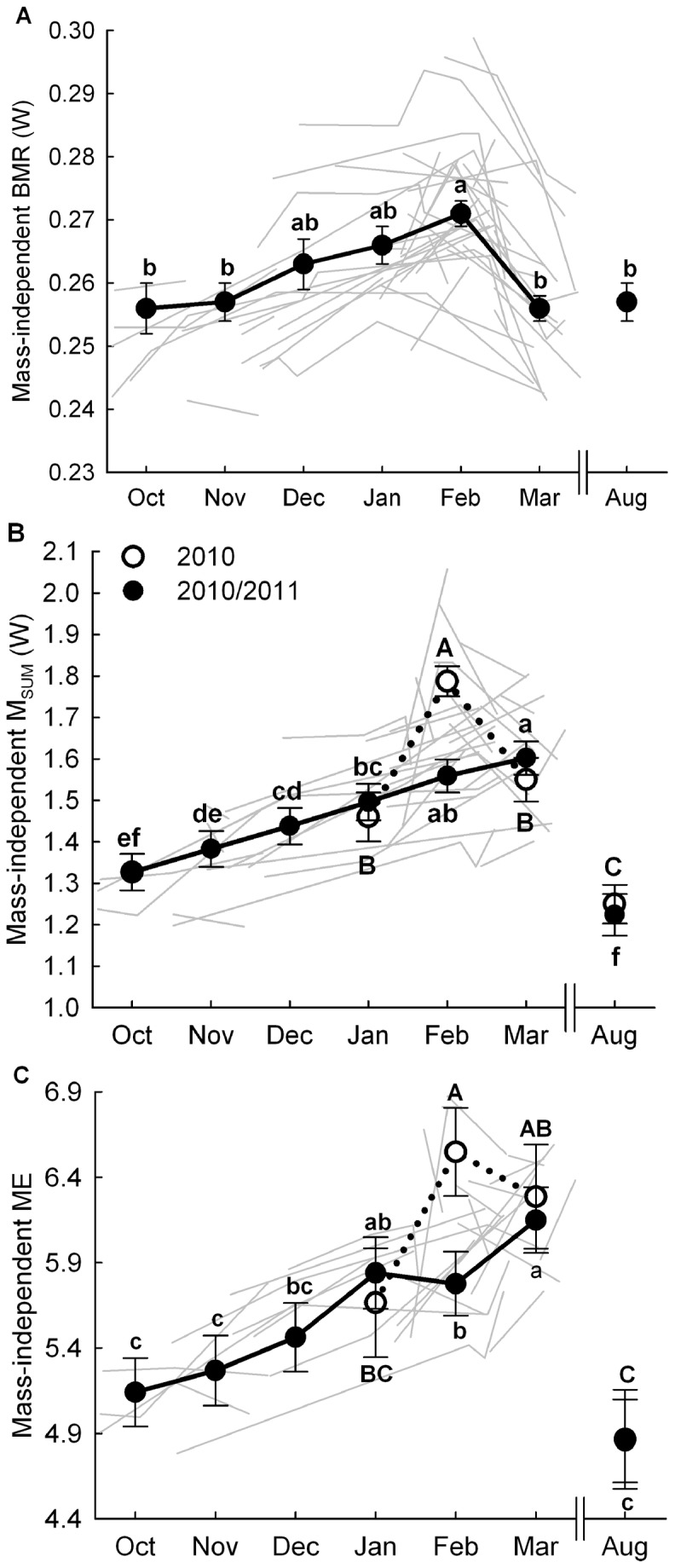
Monthly variation at the population and individual levels in mass-independent BMR, M_SUM_and ME. Population data (black line and dots) are least square means for BMR (A), M_SUM_ (B) and ME (C) controlling for year, month, sex, body mass and bird ID as random parameter. In B and C - dotted line, open dots and uppercase: 1^st^ year; solid line, black dots and lowercase: 2^nd^ year. Different letters represent significant difference between months within a year. Predicted values calculated from LMEs (see text for details) are used to visualize data from individuals captured more than once (grey lines).

Mass-independent BMR (body mass: F_1, 218_ = 83.1.2, p < 0.0001) did not change between years but varied within winter (month: F_6,321_ = 6.1, p < 0.0001, Figure 3A). BMR progressively increased by 5.9% between October and February (Tukey: p < 0.05) and then decreased by 5.5% between February and March (Tukey: p < 0.0001).

As for BMR, mass-independent M_SUM_ (body mass: F_1,204_ = 97.6, p < 0.0001) did not differ between years but varied within winter (month: F_6,270_ = 37.1, p < 0.0001). Average M_SUM_ increased by 25.0% between October and February and weakly decreased by 5.2% between February and March. However, the month effect also depended on the year (year*month: F_3,266_ = 10.0, p < 0.0001). Separated analyses by year showed that the M_SUM_ peak observed in February was only apparent in 2010, where it reached a value 31.9% higher than our intra-seasonal reference point in October, before declining by 13.3% between February and March (Tukey: p < 0.01). During the second year, mass-independent M_SUM_ increased steadily throughout winter to reach a peak in March with a value +20.7% higher than that measured in October (Tukey: p < 0.0001) (Figure 3B).

Mass-independent winter metabolic expansibility (body mass: F_1,156_ = 8.2, p < 0.01) varied with month (F_6,253_ = 18.3, p < 0.0001) with average mass-independent ME increasing by 19.8% between October and March. However, the month effect was also dependent on the year (year*month: F_3,253_ = 4.9, p < 0.01). Separated analyses revealed that during the first year, mass-independent ME reached a peak (6.5 x BMR) in February (+27.0% relative to October 2011) before decreasing non-significantly in March (-2.6%, Tukey: p = 0.8). During the second year, mass-independent ME reached its highest value (6.2 x BMR) in March (+19.6% relative to October, Tukey: p < 0.0001) (Figure 3C).

For all metabolic parameters, visual inspection of predicted values for recaptured individuals showed a high level of variability between birds but consistency in their position relative to others (i.e. there were “high” and “low” BMR/M_SUM_ individuals). Individual patterns were comparable to that observed at the population level (Figure 3).

### (d) Relationship between BMR and M_SUM_

Linear regression between residuals of whole BMR and whole M_SUM_ extracted from linear mixed effect models resulted in a significant but weak positive relationship between these parameters (n = 269; r^²^_adj_ = 0.04; p < 0.001). The relationship was not significant when using residuals from models controlling for body mass (p = 0.4).

## Discussion

This study is the first to document, with an extensive dataset, intra-seasonal and intra-individual patterns of change (e.g. reaction norm [9]) in metabolic performance of free-living birds wintering at northern latitudes. As expected, both BMR and M_SUM_ were higher in winter relative to summer and increased during the coldest months of winter. However, average BMR and average M_SUM_ followed dissimilar paths with BMR declining to summer level in the spring while M_SUM_ tended to remain high, resulting in the highest metabolic expansibility being recorded in March. Variations in metabolic performance observed at the population level reflected that observed within individuals.

### (a) Inter-seasonal variation in metabolic performance

Mass-independent basal metabolic rate, interpreted here as the energy expenditure of physiological systems remaining active in a resting bird, peaked at 0.27 W in wintering black-capped chickadees. This is comparable to values measured in wintering chickadees from Ohio (0.26 W) and Wisconsin (0.27W) [48] but lower than the ones measured in birds spending their winter in New York (0.29) [49], South Dakota (0.30 W) [48] and Alaska (0.42 W) [50]. It is therefore not surprising to find that the 6% seasonal increase in mass-independent BMR observed here is much lower than the +14% found by Cooper and Swanson in birds from South Dakota [12]. This seasonal variation is, however, in the relatively large range of seasonal changes observed in other temperate free-living resident passerines (from -4.3% in *Carpodacus* *mexicanus* [51] to +36.3% in *Passer* *montanus* [18]). Assuming the seasonal elevation in BMR reflects an increase in maintenance costs [17,18], it therefore appears that black-capped chickadees from our study site only face a moderate rise in maintenance energy demand in association with seasonal cold acclimatization relative to other populations. If temperature is one of the drivers of winter metabolic performance [10,23], it is likely that differences between populations reflect physiological responses to local conditions.

As for BMR, the seasonal increase in M_SUM_ (+34%) was in the range of previously reported observations. Seasonal changes in mass-independent M_SUM_ range from +16.2% in *Sitta* *carolinensis* [13,51] to +42.4% in *Picoides* *pubescens* [13], with black-capped chickadees from South Dakota showing a 26.3% increase in winter mass-independent M_SUM_ relative to summer [12].

### (b) Intra-seasonal variation in metabolic performance and the uncoupling of BMR and M_SUM_

Temperature has been suggested as one of the drivers of winter metabolic performance [10,20,23,48] and this led us to predict a gradual increase in BMR and M_SUM_ during winter where a peak would be observed during the coldest months of the season. Seasonal variations in BMR were of lower amplitude than expected but changes in average values were consistent with our prediction. Although significant differences were only clear when comparing February with October, November and March, BMR clearly tended to be higher during December, January and February, the coldest months of winter (both years combined, table 1). However, BMR declined rapidly in March, when ambient temperatures were still relatively cold (-16°C and -27°C mean minimum temperature in 2010 and 2011 respectively), which suggests that temperature may not be the sole driver of winter BMR phenotype in chickadees.

In contrast, intra-seasonal changes in average M_SUM_ differed between years. During the first winter, M_SUM_ peaked in February and declined in March while in the second year M_SUM_ increased until the end of our measurements in March. M_SUM_ variations were therefore consistent with our predictions for the first year, where February was the coldest month based on minimal temperature, but were counterintuitive for the second year where no decline in M_SUM_ were observed. It must be noted, however, that cold energy-demanding temperatures lasted much longer during the second winter since, although daily temperatures were already warming by March, minimal temperatures were as cold in March as in January. It is therefore likely that birds maintained their thermogenic capacity to its maximal level as long as days with very cold temperature prevailed. As metabolic expansibility is the ratio of M_SUM_ on BMR, and given the differences in range of intra-seasonal changes in BMR and M_SUM_, variations in ME were inevitably affected by changes in M_SUM_. It was therefore of no surprise to find similar variation when comparing ME and M_SUM_.

**Figure 4:**
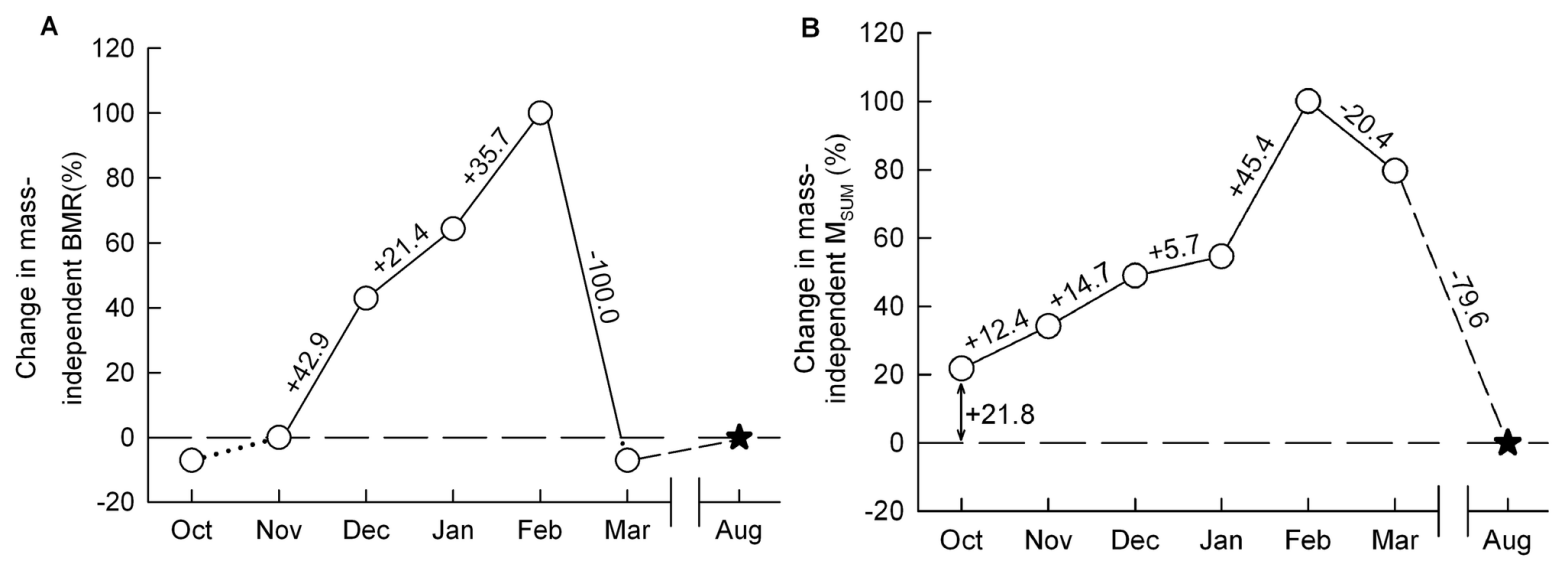
Visual representation of the monthly winter increment in metabolic performance. Increment in mass-independent BMR (A) and M_SUM_ (B) relative to summer values. Data measured in August are used as the summer phenotype reference point (August = 0%, black star, dashed line) while maximal levels of BMR and M_SUM_ recorded in winter are considered representative of the “peak” winter phenotype (February = 100%). Monthly changes in metabolic performance (values above lines) are presented in percent of total inter-seasonal difference. Dotted lines represent BMR variations below the summer reference point.

The contrast in BMR and M_SUM_ patterns goes in hand with the hypothesis that these components of metabolic performance respond to different sets of environmental constraints [20,27,28]. Comparing intra-seasonal patterns of change in BMR and M_SUM_ relative to summer values also suggests a certain level of independence between these parameters. Although it was moderate, the increase in mass-independent BMR between seasons (+5.9% comparing August and February) was the same as that detected between October and February (+5.9%). In fact, the increase in BMR started after November, when birds began to face minimal temperatures below -10°C (table 1), and remained relatively steady until February (Figure 4A). In contrast, the inter-seasonal change in mass-independent M_SUM_ (+34.2% between August and February) was higher than that measured within season (+25.0% between October and February). In fact, M_SUM_ had already achieved 21.8% of its inter-seasonal increase when we began our measurements in October (Figure 4B) meaning that, although the largest change in M_SUM_ appeared between January and February (+45.4% of total inter-seasonal increase, Figure 4B), this parameter began to change well before the beginning of sub-zero mean ambient temperatures (table 1). These results therefore suggest that flexible adjustments in thermogenic capacity appear relatively early in autumn (before October) while physiological components reflected in BMR only begin to change later (from November).

Why would BMR begin to increase later in autumn and decline earlier in the spring than M_SUM_? The reasons for the uncoupling of these variables are not clear. However, if one considers M_SUM_ as a variable mainly influenced by the size of shivering muscles [21,31,32,51] and BMR as reflecting changes in size and activity of the main digestive organs in response to cold acclimatization [18,52,53] then it is likely that those metabolic parameters reflect different sets of physiological constraints acting relatively independently, both being associated with winter [54]. Pectoral muscles would start changing early in the season in association with the appearance of cold ambient temperatures (note that sub-zero minimal temperatures were already recorded in October), whereas winter BMR variations would likely reflect changes in diet and in the amounts of food consumed (to sustain an increasing daily energy expenditure, DEE). Chickadees feed mainly on insects during summer but eat up to 50% vegetal matter during winter [55] while food supplementation by feeders may represent only up to 21% of their daily energy intake [56]. Snow typically starts to fall in November at our field site while insects are already visible by March. Therefore, it could be that the combination of a winter increase in DEE and changes in natural diet digestibility and energy content leads to a restructuring of the digestive apparatus, and in turn changes in BMR, appearing independently from those observed in M_SUM_. The proximal effect of cold ambient temperature on parameters of metabolic performance [10,23,48] would therefore be much more influential for M_SUM_ than for BMR. Experimental research is needed to test this “metabolic uncoupling” hypothesis and to determine what biotic and/or abiotic factors trigger seasonal changes in parameters of metabolic performance.

Given the seasonal mismatch in variations of BMR and M_SUM_, it is therefore of no surprise to find a lack of significant correlation between these parameters when controlling for body mass in regression analyses. Independence of BMR and M_SUM_ has also been observed by others [15,27,28,57], but findings remains conflicting [25,33]. Experimental manipulations of BMR and M_SUM_, for example by combining diet and temperature treatments, should therefore be conducted to confirm findings.

### (c) Intra-individual variation in winter metabolic performance

Studies on seasonal variation of avian metabolic performance are typically conducted at the population level [11,18,30,51] and, although it is rarely stated, they generally assume that population patterns are reflective of those observable within individuals. As far as we know, this is the first study to document with an extensive dataset seasonal variation of metabolism at both the population and individual levels in a resident bird species. Our findings support the common assumption; patterns observed at the population level reflected intra-individual variation in body mass, mass-independent BMR, M_SUM_ and ME (Figure 3A–C) and are therefore representative of average individual phenotypic flexibility.

### (d) Is metabolic expansibility a meaningful variable?

In this study, we considered metabolic expansibility as an indicator of the capacity of an organism to produce heat (because it was based on M_SUM_) for a given size of metabolic machinery (which would be reflected by BMR) [12,15,26,28,34]. However, the lack of correlation between BMR and M_SUM_, as observed by others [15,27,28,57], as well as the temporal mismatch in these parameters during seasonal acclimatization suggest a lack of a functional relationship between BMR and M_SUM_. Although BMR includes the resting energy consumption of physiolgical components involved in active thermogenesis (i.e. muscles), evidences suggest that, in the context of cold acclimatization, its variations are mainly influenced by activity and size of organ systems involved in energy acquisition and digestion [18,30,52], while M_SUM_ would mostly reflect active energy consumption of shivering muscles [21,31,32]. It therefore becomes apparent to us that metabolic expansibility, as we defined it earlier, may lead to misleading conclusions about machinery adjustments. We therefore suggest caution with the interpretation of this variable and recommend interpreting variations in BMR and M_SUM_ separately to infer on animals metabolic capacity.

## Supporting Information

Table S1 Inter-seasonal variation in body mass, BMR, M_SUM_ and ME. Data are least square means controlling for year, season and sex (and time of capture for body mass) with bird ID as random parameter.
(DOC)

Table S2 Intra-seasonal variation in body mass, BMR, M_SUM_ and ME. Data are least square means controlling for sex, year, month and the interaction year*month for M_SUM_ and ME, with bird ID as random parameter. Body mass analysis also included time of capture as covariate.
(DOC)
