# Supplementary material for: Intra-Seasonal Flexibility in Avian Metabolic Performance Highlights the Uncoupling of Basal Metabolic Rate and Thermogenic Capacity
Source: PLoS One. 2013 Jun 28;8(6):e68292. doi: 10.1371/journal.pone.0068292 (PMC3695913; doi:10.1371/journal.pone.0068292)
Supplement: Table S1 — Data are least square means controlling for year, season and sex (and time of capture for body mass) with bird ID as random parameter. (DOC) [file pone.0068292.s001.doc]

Table S1 : Inter-seasonal variation in body mass, BMR, M_SUM_ and ME. Data are least square means controlling for year, season and sex (and time of capture for body mass) with bird ID as random parameter.

|  | Body mass (g) | BMR (W) | M_SUM_ (W) | ME |
| --- | --- | --- | --- | --- |
| Seasons | mean ± sem | mean ± sem | mean ± sem | mean ± sem |
| Winter | 11.44 ± 0.06 | 0.271 ± 0.002 | 1.667 ± 0.065 | 6.19 ± 0.25 |
| Summer | 11.42 ± 0.08 | 0.253 ± 0.004 | 1.241 ± 0.071 | 4.99 ± 0.28 |
