# Supplementary material for: Intra-Seasonal Flexibility in Avian Metabolic Performance Highlights the Uncoupling of Basal Metabolic Rate and Thermogenic Capacity
Source: PLoS One. 2013 Jun 28;8(6):e68292. doi: 10.1371/journal.pone.0068292 (PMC3695913; doi:10.1371/journal.pone.0068292)
Supplement: Table S2 — Data are least square means controlling for sex, year, month and the interaction year*month for MSUM and ME, with bird ID as random parameter. Body mass analysis also included time of capture as covariate. (DOC) [file pone.0068292.s002.doc]

**Table S1 :** Intra-seasonal variation in body mass, BMR, M_SUM_ and ME. Data are least square means controlling for sex, year, month and the interaction year*month for M_SUM_ and ME, with bird ID as random parameter. Body mass analysis also included time of capture as covariate.

|  | Body mass (g) | BMR  (W) | M_SUM_ (W)  year 1 | M_SUM_ (W) year 2 | ME  year 1 | ME  year 2 |
| --- | --- | --- | --- | --- | --- | --- |
| Months | mean ± sem | mean ± sem | mean ± sem | mean ± sem | mean ± sem | mean ± sem |
| Oct | 11.12 ± 0.08 | 0.256 ± 0.004 |  | 1.316 ± 0.049 |  | 5.142 ± 0.200 |
| Nov | 11.03 ± 0.07 | 0.252 ± 0.004 |  | 1.339 ± 0.049 |  | 5.268 ± 0.205 |
| Dec | 11.56 ± 0.07 | 0.264 ± 0.004 |  | 1.460 ± 0.049 |  | 5.463 ± 0.201 |
| Jan | 11.38 ± 0.06 | 0.264 ± 0.003 | 1.483 ± 0.093 | 1.494 ± 0.049 | 5.666 ± 0.319 | 5.840 ± 0.209 |
| Feb | 11.44 ± 0.05 | 0.271 ± 0.002 | 1.782 ± 0.080 | 1.555 ± 0.044 | 6.549 ± 0.258 | 5.777 ± 0.187 |
| Mar | 11.35 ± 0.05 | 0.254 ± 0.003 | 1.555 ± 0.089 | 1.572 ± 0.046 | 6.287 ± 0.305 | 6.150 ± 0.192 |
| Aug | 11.32 ± 0.07 | 0.255 ± 0.004 | 1.274 ± 0.085 | 1.191 ± 0.057 | 4.865 ± 0.290 | 4.856 ± 0.243 |
